# *Notes from the Field:* Geo-Temporal Trends in Fentanyl Administration Routes Among Adults Reporting Use of Illegally Manufactured Fentanyl When Assessed for Substance-Use Treatment — 14 U.S. States, 2017–2023

**DOI:** 10.15585/mmwr.mm7350a4

**Published:** 2024-12-19

**Authors:** Yijie Chen, Xinyi Jiang, R. Matthew Gladden, Nisha Nataraj, Gery P. Guy, Deborah Dowell

**Affiliations:** 1 Division of Overdose Prevention, National Center for Injury Prevention and Control, CDC.

**Summary**

**What is already known about this topic?**

From 2020 to 2022, among overdose deaths with only illegally manufactured fentanyl (IMF) detected, those with evidence of smoking IMF increased by 78.9%, and those with evidence of injection decreased by 41.6%.

**What is added by this report?**

From July–December 2017 to January–June 2023, the percentage of persons injecting IMF sharply declined across all U.S. Census Bureau regions, with region-specific differences in magnitude; correspondingly, IMF snorting or sniffing increased in the Northeast, and IMF smoking increased in the Midwest, South, and West regions.

**What are the implications for public health practice?**

Whereas avoiding injection likely reduces infectious disease transmission, noninjection routes might still contribute to overdose. Provision of locally tailored messaging and linkage to medical treatment is important among persons using IMF through noninjection routes.

During 2019–2023, U.S. overdose deaths involving fentanyl have more than doubled, from an estimated 35,474 in 2019 to 72,219 in 2023 (*1*). From 2020 to 2022, overdose deaths with only illegally manufactured fentanyl (IMF) detected and evidence of smoking IMF increased by 78.9%; deaths with evidence of injection decreased by 41.6% (*2*). Smoking, however, could not be linked specifically to IMF use when deaths involved multiple drugs (e.g., methamphetamine co-used with IMF). To characterize IMF administration routes among all persons who use IMF, with or without other drugs, IMF administration routes were examined among adults assessed for substance use treatment who used IMF during the past 30 days.

## Investigation and Outcomes

The National Addictions Vigilance Intervention and Prevention Program’s Addiction Severity Index-Multimedia Version (ASI-MV) tool* includes a convenience sample of adults aged ≥18 years assessed for substance-use treatment. CDC analyzed treatment assessments conducted between July 1, 2017, and June 30, 2023, which were restricted to 14 states^†^ with at least 100 assessments reporting past 30-day IMF use (16,636)^§^ and stratified by administration routes (swallowed, snorted or sniffed, smoked, and injected^¶^). The percentage of persons reporting each administration route was calculated for 6-month periods by U.S. Census Bureau region.** Significant (p-value <0.05) trends by administration route were identified using Joinpoint (Joinpoint version 5.1.0; National Cancer Institute) and Pearson correlations. This activity was reviewed by CDC, deemed not research, and was conducted consistent with applicable federal law and CDC policy.^††^

In the Midwest, South, and West U.S. Census Bureau regions, increases in smoking (from 7.8% during July–December 2017 to 38.2% during January–June 2023 [Midwest]; from 15.4% during January–June 2020 to 54.0% during January–June 2023 [South]; and from 45.7% during January–June 2018 to 85.7% during January–June 2023 [West]) were strongly negatively correlated with decreases in injection (Pearson correlation coefficient [r] = −0.96; p<0.001 [Midwest]; −0.98; p<0.001 [South]; and −0.74; p<0.01 [West]). Injection decreased from 75.2% during January–June 2020 to 41.2% during January–June 2023 in the Midwest U.S. Census Bureau region; from 54.2% during July–December 2020 to 30.3% during January–June 2023 in the South; and from 65.6% during July–December 2018 to 9.1% during January–June 2023 in the West, but timing of changes across each census region varied (Figure). In the Northeast, increases in snorting or sniffing (from 18.9% during July–December 2017 to 45.5% during January–June 2023) were strongly negatively correlated (r = −0.89; p<0.001) with a decrease in injection (from 83.8% during July–December 2017 to 63.4% during January–June 2023).

**FIGURE:**
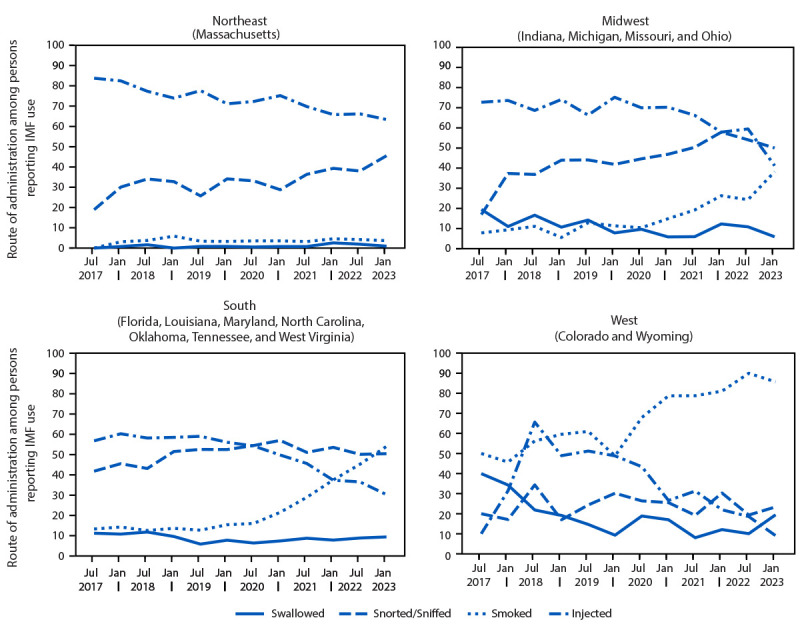
Routes of administration* of illegally manufactured fentanyl among persons assessed for substance use treatment, by U.S. Census Bureau region^†^ — 14 states, July 2017–June 2023 **Abbreviation**: IMF = illegally manufactured fentanyl. * IMF administration routes are not mutually exclusive. A person can report more than one route in the survey, and the sum of proportions can therefore exceed one. Injection includes injection into the vein or into the skin or muscle. ^†^ Selected states with at least 100 assessments reporting past 30-day IMF use during July 2017–June 2023: *Northeast*: Massachusetts (3,692); *Midwest*: Michigan (1,026), Missouri (463), Indiana (186), and Ohio (130); *South*: Tennessee (6,200), Oklahoma (2,201), Maryland (1,248), Florida (250), Louisiana (198), West Virginia (127), and North Carolina (123); and *West*: Wyoming (409) and Colorado (383).

## Preliminary Conclusions and Actions

Consistent with other fatal overdose investigations (*2*), the percentage of persons injecting IMF sharply declined across all U.S. Census Bureau regions between 2017 and 2023, although the magnitudes of these declines were region-specific. Some persons who use IMF reportedly believe that smoking is safer than injecting IMF (*3*). Whereas avoiding injection likely reduces the risk for acquiring bloodborne viruses (e.g., HIV or HCV) and soft tissue infections (*2*,*4*), noninjection routes might contribute to overdose or other health problems (e.g., orofacial lesions associated with snorting) (*5*). Compared with injection, smoking IMF is associated with a higher frequency of use throughout the day and potentially higher daily dosages consumed (*3*). Substantial shifts to smoking IMF in the Midwest, South, and West, and sniffing or snorting IMF in the Northeast (i.e., Massachusetts) highlight the need to understand local trends in drug use and tailor local messaging, outreach, and linkage to medical care, including effective treatment for opioid use disorder in persons using IMF through noninjection routes.

## Footnotes

*
https://uprisehealth.com/resources/national-addictions-vigilance-intervention-and-prevention-program-navipprotm-a-real-time-product-specific-public-health-surveillance-system-for-monitoring-prescription-drug-abuse/

† Selected states with at least 100 assessments reporting past 30-day IMF use during July 2017–June 2023: *Northeast*: Massachusetts (3,692); *Midwest*: Michigan (1,026), Missouri (463), Indiana (186), and Ohio (130); *South*: Tennessee (6,200), Oklahoma (2,201), Maryland (1,248), Florida (250), Louisiana (198), West Virginia (127), and North Carolina (123); and *West*: Wyoming (409) and Colorado (383).

§ The ASI-MV assessment asks persons completing the questionnaire, “In the past 30 days, estimate how many days you used street fentanyl.” The definition of street fentanyl in the ASI-MV was “Street fentanyl (illegal fentanyl and carfentanil, sometimes combined with other drugs such as heroin or cocaine).” In this study, 2.6% of ASI-MV assessments were repeat assessments, indicating that they were completed by a unique person with a previous assessment within the 6-month period.

¶ IMF administration routes are not mutually exclusive. A person can report more than one route in the survey, and the sum of proportions can exceed one. The injection category includes injection in the vein and the skin or muscle.

** U.S. Census Bureau regions were used to stratify jurisdictions into geographic regions (https://www2.census.gov/geo/pdfs/maps-data/maps/reference/us_regdiv.pdf). Region analysis included one of nine jurisdictions in the Northeast region, four of 12 jurisdictions in the Midwest region, seven of 17 jurisdictions in the South region, and two of 13 jurisdictions in the West region.

†† 45 C.F.R. part 46, 21 C.F.R. part 56; 42 U.S.C. Sect. 241(d); 5 U.S.C. Sect. 552a; 44 U.S.C. Sect. 3501 et seq.

## References

[1] Ahmad FB, Cisewski JA, Garnett M, Warner M. National Vital Statistics Program: provisional drug overdose death counts by specific drugs. Hyattsville, MD: US Department of Health and Human Services, CDC, National Center for Health Statistics; 2024. https://www.cdc.gov/nchs/nvss/vsrr/prov-drug-involved-mortality.htm

[2] Tanz LJ, Gladden RM, Dinwiddie AT, Routes of drug use among drug overdose deaths—United States, 2020–2022. MMWR Morb Mortal Wkly Rep 2024;73:124–30. 10.15585/mmwr.mm7306a2 38358969 PMC10899081

[3] Ciccarone D, Holm N, Ondocsin J, Innovation and adaptation: the rise of a fentanyl smoking culture in San Francisco. PLoS One 2024;19:e0303403. 10.1371/journal.pone.0303403 38776268 PMC11111043

[4] Megerian CE, Bair L, Smith J, Health risks associated with smoking versus injecting fentanyl among people who use drugs in California. Drug Alcohol Depend 2024;255:111053. 10.1016/j.drugalcdep.2023.111053 38128362

[5] Gold M, Boyack I, Caputo N, Pearlman A. Imaging prevalence of nasal septal perforation in an urban population. Clin Imaging 2017;43:80–2. 10.1016/j.clinimag.2017.02.002 28242555

